# Discrepancies between the Spatial Distribution of Cancer Incidence and Mortality as an Indicator of Unmet Needs in Cancer Prevention and/or Treatment in Hungary

**DOI:** 10.3390/cancers16162917

**Published:** 2024-08-22

**Authors:** Róza Ádány, Attila Juhász, Csilla Nagy, Bernadett Burkali, Péter Pikó, Martin McKee, Beatrix Oroszi

**Affiliations:** 1 Epidemiology and Surveillance Centre, Semmelweis University, 25. Üllői Street, 1085 Budapest, Hungary; juhasz.attila@semmelweis.hu (A.J.); nagy.csilla@semmelweis.hu (C.N.); burkali.bernadett@semmelweis.hu (B.B.); piko.peter@semmelweis.hu (P.P.); oroszi.beatrix@semmelweis.hu (B.O.); 2 Department of Public Health and Epidemiology, Faculty of Medicine, University of Debrecen, 26. Kassai Street, 4028 Debrecen, Hungary; 3 HUN-REN Public Health Research Group, University of Debrecen, 26. Kassai Street, 4028 Debrecen, Hungary; 4 Department of Preventive Medicine and Public Health, Semmelweis University, 1085 Budapest, Hungary; 5 Department of Quality Management in Healthcare and Infection Control, Petz Aladár Teaching Hospital of Győr-Moson-Sopron County, 2-4. Vasvári Pál Street, 9024 Győr, Hungary; 6 Department of Health Services Research and Policy, London School of Hygiene and Tropical Medicine, Keppel Street, London WC1E 7HT, UK; martin.mckee@lshtm.ac.uk

**Keywords:** cancer incidence, cancer mortality, spatial inequalities, socioeconomic, deprivation, disease mapping

## Abstract

**Simple Summary:**

Socioeconomic inequalities in cancer incidence and mortality have been studied extensively, but there has been no investigation of the spatial distribution of incidence and mortality within a country to see if they are consistent with each other and with deprivation. To fill this gap, we examined the spatial distribution of cancer incidence and mortality and their association with each other and with socioeconomic deprivation at the municipal level in Hungary, one of the countries with the most unfavourable cancer burden in the world. For different types of cancer, while mortality always showed positive associations with deprivation, there was only partial overlap between areas of high incidence and mortality across the country, with clusters often independent of deprivation. Even where incidence and mortality overlapped geographically, there were often significant differences in mortality risk. Discrepancies in the spatial distribution of cancer incidence and mortality are indicators of an unmet need for cancer prevention and/or treatment.

**Abstract:**

There is a rich body of literature on the distribution of cancer incidence and mortality in socioeconomically different world regions, but none of the studies has compared the spatial distribution of mortality and incidence to see if they are consistent with each other. All malignant neoplasms combined and cervical, colorectal, breast, pancreatic, lung, and oral cancers separately were studied in the Hungarian population aged 25–64 years for 2007–2018 at the municipality level by sex. In each case, the spatial distribution of incidence and mortality were compared with each other and with the level of deprivation using disease mapping, spatial regression, risk analysis, and spatial scan statistics. A positive association between deprivation and mortality was found for each type of cancer, but there was no significant association for male colorectal cancer (relative risk (RR) 1.00; 95% credible interval (CI) 0.99–1.02), pancreatic cancer (RR: 1.01; 95%CI 0.98–1.04), and female colorectal cancer incidence (RR: 1.01; 95%CI 0.99–1.03), whereas a negative association for breast cancer (RR: 0.98; 95%CI 0.96–0.99) was found. Disease mapping analyses showed only partial overlap between areas of high incidence and mortality, often independent of deprivation. Our results highlight not only the diverse relationship between cancer burden and deprivation, but also the inconsistent relationship between cancer incidence and mortality, pointing to areas with populations that require special public health attention.

## 1. Introduction

Cancer is among the leading contributors to disease burden worldwide [1]. The incidence of cancer in the European Union is increasing, and it increased by 2.3% between 2020 and 2022 to reach 2.74 million. Cancer deaths increased slightly more over this period, by 2.4%, to reach 1.29 million [2].

It is well known that, with a few exceptions such as breast cancer, cancer incidence is higher in populations with greater socioeconomic deprivation (measured in various ways including composite indices, education, income, employment, etc.), reflecting, for example, greater exposure to risk factors and delayed detection and treatment of pre-malignant lesions or early-stage malignancies. 

Once cancer has developed, disadvantaged individuals and populations may experience poorer outcomes [3], although this varies by cancer site and is mediated, to some extent, by different levels of comorbidities [4]. This reflects factors such as late presentation and delayed or less effective treatment and is especially so for the most treatable cancers [5].

A comprehensive policy response to cancer that emphasises the importance of reducing these well-known socioeconomic inequalities should include an analysis of both incidence and outcome. As many aspects of deprivation are geographically clustered, it may seem reasonable to assume that patterns of high incidence of many cancers and poor outcomes would be geographically correlated; we argue that it cannot be assumed that they are. However, we are not aware of any published studies that have investigated this. 

However, there are challenges in doing so. The best measure of outcome for people with cancer is survival, but this is only available in those countries that have cancer registries that cover the entire population, with record linkage that allows them to follow individuals through their therapeutic journey to either survival at a defined point in time (usually one or five years) or death. Ideally, such data would provide information on both the individual’s socioeconomic status and the level of deprivation in the area in which they live as the influence of these factors may differ [6]. Unfortunately, relatively few countries collect data on survival, either because the registries do not cover the entire population or because they cannot link individuals’ records across databases. However, they often collect data on cancer mortality in the population. 

Clearly, there is an imperative to create cancer registries that can provide such data, but, where they do not exist, policymakers may be able to gain some insights by comparing socioeconomic patterns and spatial clustering of incidence and mortality. As noted above, this is pragmatic rather than ideal. Since mortality is a function of both incidence and survival, the relative contributions are unknowable, so it is important to avoid anything that would involve calculating mortality–incidence ratios, which are known to be both theoretically and empirically inappropriate [7].

To inform those engaging in such analyses, we ask whether the associations between deprivation and mortality are similar for particular cancers and whether the areas with high incidence are the same as those with high mortality. The answers to these questions provide a starting point for a more detailed investigation. 

We do this using data from Hungary. The rationale for choosing Hungary is, first, that it has high-quality data on both incidence and mortality, allowing fine spatial disaggregation down to the level of municipalities for which data on deprivation levels are available, and second, that it has a very high cancer burden. Although the Hungarian cancer registry collects some data on survival, they was only available for a minority of cases in a recent study [8]. In addition, Hungary ranked first among WHO Europe and Organisation for Economic Co-operation and Development (OECD) countries in 2022 and third (after Mongolia and Zimbabwe) among the 185 countries monitored by GLOBOCAN, largely due to the very high mortality from lung, pancreatic, and colorectal cancer [1]. As the latest OECD “Country Cancer Profile 2023 Hungary” [9] points out, cancer care is centralised in county, regional, and national centres. All 19 counties have county centres, which are responsible for the provision of care for high-incidence cancers. These centres work in a multidisciplinary manner, and 14 also operate as radiotherapy centres. The report concludes that “However, efforts to measure cancer care quality have not been consistent nationally and are still limited. A systematic clinical audit system is yet to be implemented. Also, collecting performance measures on outcomes and care experiences from the perspective of people with cancer is in its infancy.” The report also emphasises that in the Hungarian healthcare system, all elements of cancer care—from screening to palliative care—are generally available through public financing. However, inequalities persist, both in the early detection and treatment of cancer. A shortage of cancer care specialists persists at the regional and county levels, combined with a low density of radiation therapy equipment, which contributes to geographical disparities in access to care. In addition, Hungary spent EUR 226 per capita on cancer care, which is only 69% of the EU average.

The Hungarian government has instituted nationwide population-based screening programmes (screening offered to a specific at-risk target population) for three types of cancers. The breast cancer screening programme started in 2001 and targets women aged 45–65 years. The programme for cervical cancer started in 2003 and targets women aged 25–65 years. In 2024 (not the first time), a screening programme was introduced for colorectal cancer for those aged 50–70 years. The most recently completed HUNCHEST-II project conducted in 18 medical facilities demonstrated that low-dose CT screening for lung cancer facilitates early diagnosis, thus arguing in favour of introducing systematic LC screening in Hungary [10]. The OECD “Health at a glance 2023” report [11] also underlines that the participation rate in the presently operating screening programs is not sufficient in Hungary (mammography screening 30%, cervical screening 26%, and especially low—only 3%—in the case of colorectal cancer screening program). A new regulation (Act XXIX of 2024, § 31), which will come into force on 1 January 2025, will make these screening tests mandatory. If an individual refuses to undergo the screening test, the public health administration will issue an immediately enforceable decision, the specifics of which have yet to be determined. In addition to the ethical and privacy concerns that the regulation raises, its efficacy from a healthcare management perspective is also debatable.

## 2. Materials and Methods

### 2.1. Data

The spatial distribution of incidence and mortality from all malignant neoplasms (ICD-10.:C00-C97, D05-D06, excluding C44) and separately for certain common cancer sites. The sites were selected to include three with a 5-year survival of over 50% (using data from England, which has data covering the entire population with a high-quality data linkage) [12] and two where survival is low. The former comprise malignant neoplasms of the cervix uteri (ICD-10.:C53, D06), of the colon, rectum, and anus (ICD-10.:C18-C21), and of the breast (ICD-10.:C50, D05). The latter were malignant neoplasms of the pancreas (ICD-10.:C25) and those of the trachea, bronchus, and lung (ICD-10.:C33-C34)—for both of which Hungary ranks first in the world [1]. The 5-year survival percentages for these sites, using the English data, are 61%, 57%, 86%, 7%, and 17%, respectively. We analysed data covering the entire Hungarian population aged 25–64 years from 2007 to 2018 at the municipal level, disaggregated by sex. 

The same analysis was carried out for malignant neoplasms of the lip, oral cavity, and pharynx (ICD-10.:C00-D14). In this case, survival varies considerably, depending on the site, with Cancer Research UK quoting a range of 19–59%. We have included it because it is particularly common in Hungary [13].

In each case, the spatial distribution of incidence and mortality in the 3178 Hungarian municipalities (average population 3.068) was compared with the other cases and with the level of socioeconomic deprivation for the years 2007–2018. In the rest of the paper, we also refer to Budapest and the 19 Hungarian counties, where appropriate.

Mortality data (by municipality, by year, by sex, by five-year age group) over this period were obtained from the Hungarian Central Statistical Office, incidence data (by postcode, by year, by sex, by five-year age group) were obtained from the Hungarian Cancer Registry, and population data were provided by the Deputy State Secretariat for the Management of Registers, Ministry of the Interior.

### 2.2. Deprivation Index Calculation

The area-based Deprivation Index (DI) values at the municipal level were derived from data from the most recent census, certified in Hungary, in 2011. The method of calculating the DI has been described previously [14] and successfully used in several studies [15,16]. Briefly, it uses data on seven socioeconomic indicators at the municipal level (income, educational level, unemployment rate, proportion of single-parent families, proportion of large families, density of housing, and car ownership). The variables were transformed using the natural log transformation and standardisation (z-scores). The area-specific index is a weighted sum of the z-scores, with higher values representing greater deprivation. The weight of each variable was determined based on the standardised scoring coefficients using principal component analysis. All municipalities included in the analysis were classified into five groups or quintiles, ranging from the least deprived (quintile I) to the most deprived (quintile V). Areas with positive values have a lower socioeconomic status compared to the national average, while the opposite is true for areas with negative values.

### 2.3. Disease Mapping

Spatial inequalities in adult premature mortality (i.e., in the age group of 25–64 years) and incidence (in the same age group) were investigated and described by mapping the distribution of mortality and incidence by spatial distribution. Hierarchical Bayesian estimation (BYM models) were used to estimate mortality and incidence using smoothed standardised ratios [17]. These were estimated using the “disease mapping” tool of the Rapid Inquiry Facility (RIF) software (version 3.2) [18] and the R-INLA software package (version 23.05.30) [19].

Uncertainty was estimated by determining the posterior probability, i.e., the probability that the mortality or incidence in a given area differs from the national risk, which is the reference. Areas where this probability is high (greater than 0.8) or low (less than 0.2), indicating a reasonable estimate that the event is significantly higher or lower than the national average, are marked on maps.

### 2.4. Ecological Regression

The association of deprivation with the spatial distribution of cancer incidence and mortality (for 25–64 years) was also assessed using a hierarchical Bayesian estimation model with spatial regression analysis [20]. As an extension of the BYM model [17] used in the spatial association analysis, DI was included in the model. Relative risk shows the magnitude and direction of the change in mortality/incidence associated with a unit change in DI. Estimation was performed using the R-INLA software package (version 23.05.30) [19].

### 2.5. Risk Analysis

The RIF risk analysis tool was also used to calculate the association between deprivation and cancer incidence and mortality (for 25–64 years) in Hungary. The indirectly standardised mortality ratio and the incidence ratios were calculated according to the deprivation quintiles of the municipalities. Indirect standardisation compared observed cases by sex and age group with expected events based on Hungarian mortality rates. Chi-squared tests for homogeneity and for linear trends were used to test the associations [18].

### 2.6. Proportional Model

The proportional mortality model was used as a method of jointly modelling incidence and mortality (for 25–64 years) in a way that highlights their differences by odds ratios calculated [21]. Thus, common and divergent trends between incidence and mortality are highlighted in a map of spatial random effects. The log ratio of expected counts is included as an offset term. A high value indicates that the risk of incidence and mortality differs significantly between areas in terms of the spatial component, with a higher risk of mortality as would expected based on incidence [21]. Estimation was performed using the R-INLA software package (version 23.05.30) [19].

### 2.7. Spatial Scan Statistic

Age-adjusted incidence and mortality clusters of increased risk were identified using spatial scan statistics [22] (directly linked from RIF to SaTScan version 9 [23]) to assess the location and magnitude of clusters. The age adjustment was performed using the expected number of cases for each area based on age-specific mortality rates in Hungary. The maximum spatial cluster size was set to 50% of the population at risk. The statistical significance of the most likely clusters was obtained through Monte Carlo hypothesis testing. Only significant clusters (*p* < 0.05) are shown in the maps.

## 3. Results

### 3.1. Incidence and Mortality of Malignant Neoplasms

In the Hungarian population aged 25–64, there were about half a million incident cases (males: 230,771; females: 242,726) of malignant neoplasms between 2007 and 2018, with 85,968 deaths in males and 57,094 deaths in females.

The incidence of malignant neoplasms (excluding C44) showed a modestly increasing trend for females and a slightly decreasing trend for males between 2007 and 2018. Mortality from malignant neoplasms decreased for both sexes, with a steeper decrease for males over the period studied.

**Figure 1:**
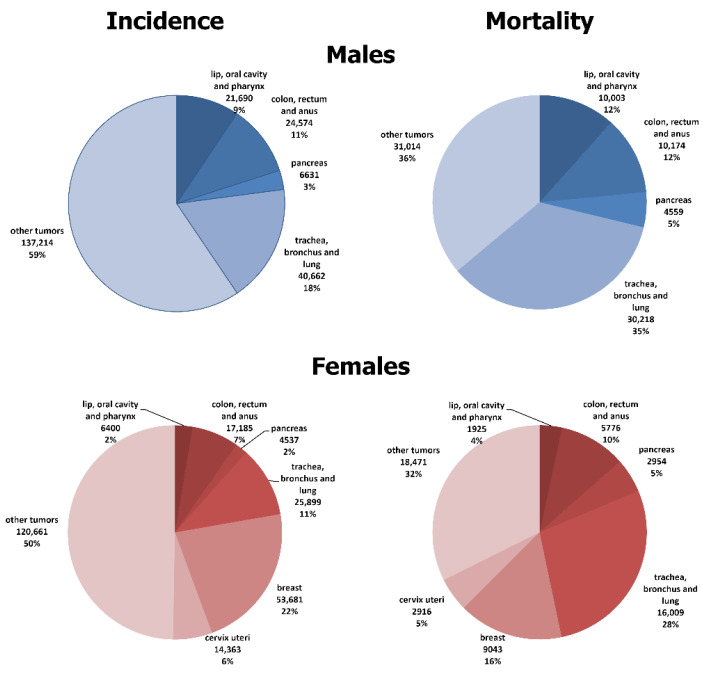
Proportion of incidence and premature mortality due to selected major malignant neoplasms in the Hungarian population, at ages 25–64, 2007–2018.

The contributions of the studied cancers to overall incidence and mortality in the 25–64 age group, by sex, are shown in Figure 1. Among males, the figures were similar for cancers of the lip, oral cavity, pharynx, and colorectum (incidence: 9% and 11%; mortality: 12% and 12%, respectively) but pancreatic and lung cancers made a greater contribution to all cancer mortality (incidence: 3% and 18%; mortality 5% and 35%, respectively). The opposite was true for females; the mortality proportion was higher than the incidence proportion for all selected cancers, except for breast and cervix uteri.

### 3.2. Spatial Variability in Deprivation

**Figure 2:**
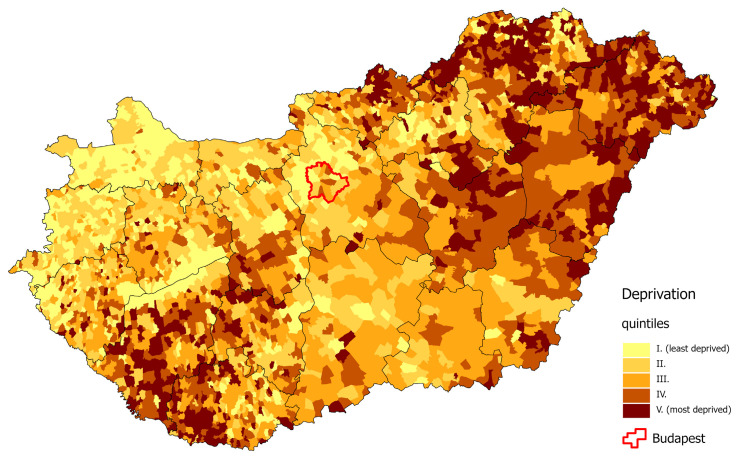
Spatial distribution of deprivation in Hungary, at the municipality level, 2011.

As described in our previous publications [24,25], the DI values of the municipalities varied from −4.9 to +8.00. We divided these values into quintiles: −4.9 ≤ DI ≤ −1.13, with an average of −1.72 (quintile I); −1.13 < DI ≤ −0.43, with an average of −0.78 (quintile II); −0.43 < DI ≤ 0.22, with an average of −0.12 (quintile III); 0.22 < DI ≤ 1.06, with an average of 0.62 (quintile IV); and 1.06 < DI ≤ 8.00, with an average of 2.02 (quintile V). The most deprived counties in 2011 were located in the north-eastern and south-western parts of Hungary. The least deprived districts in the country were located in the north-western part of Hungary, in the capital city of Budapest, and in its neighbouring areas (Figure 2) [25].

### 3.3. Association between Deprivation and Cancer Mortality and Incidence for People Aged 25–64 Years

There is a positive significant association between deprivation and both incidence and mortality of all malignant neoplasms for both sexes (Table 1 and Table 2), which increases linearly when calculated by deprivation quintile (Supplementary Tables S1 and S7).

This association was strongest for lung cancer and oral cavity cancer (Tables S1 and S2), with incidence and mortality increasing with greater deprivation (Supplementary Tables S2, S5, and S7).

For colorectal cancer, the association with deprivation was positive and significant only for mortality, and for both sexes. There was only a weakly significant positive association with incidence in men but not in women (Supplementary Tables S3 and S7).

For cervical cancer, there was a significant positive association between deprivation and both incidence and mortality (Table 1 and Table 2; Supplementary Tables S4 and S7).

In contrast, but as expected, higher deprivation was associated with lower breast cancer incidence (Table 1), but the coefficient was reversed for mortality (Table 2), although there was no clear pattern when ranked by deprivation quintile (Supplementary Tables S4 and S7).

The association between deprivation and pancreatic cancer incidence was also positive, but significant only for females (Tables S1 and S2), although the patterns were less clear when ranked by deprivation quintile (Supplementary Tables S6 and S7).

### 3.4. Results of Disease Mapping

**Figure 3:**
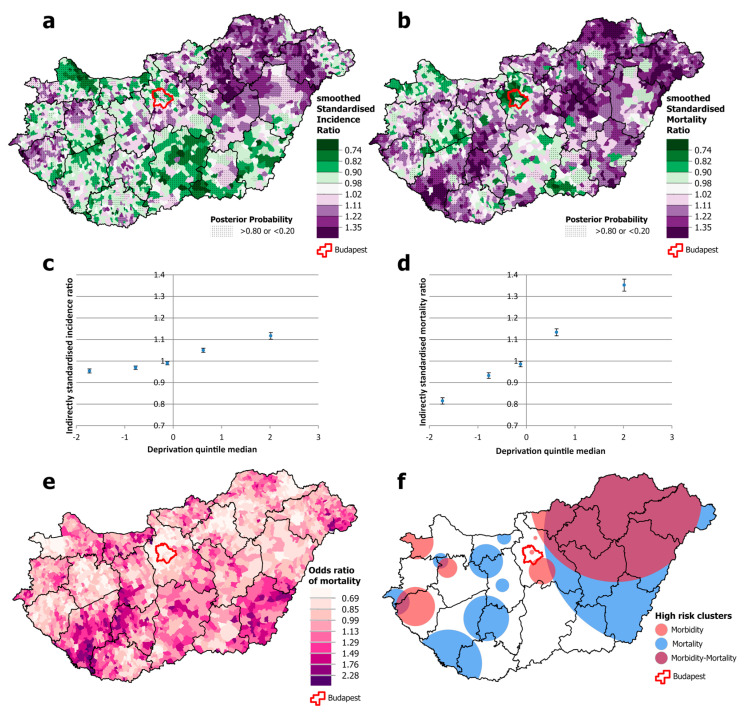
Spatial distribution of incidence (**a**) and mortality (**b**) at the municipality level; the relationship between deprivation and incidence (**c**) and mortality risk (**d**) by Deprivation Index quintile; odds ratio of mortality (**e**) and clusters of high incidence and mortality (**f**) due to malignant neoplasms, for males aged 25–64 years in Hungary, 2007–2018.

Among males, there was a significantly higher risk of both incidence and mortality from all malignant neoplasms combined in north-eastern Hungary, but this association was not apparent in the south-western and south-eastern parts of the country, both of which have high mortality (Figure 3a,b,e,f). Cancer incidence was disproportionately high in the western part of the country and in the capital, Budapest, compared with the relatively low mortality (Figure 3a,b,f). Both incidence and mortality were positively and significantly associated with deprivation, but the slope was greater for mortality than for incidence. Premature mortality from cancer in areas in the most deprived quintile was around 35% higher than the national average and almost 20% lower in the least deprived quintile. However, the differences in incidence are much smaller; incidence in the least deprived quintile was only a few percentage points below the national average, and in the most deprived quintile, it was just over 10 percentage points above (Figure 3c,d). 

**Figure 4:**
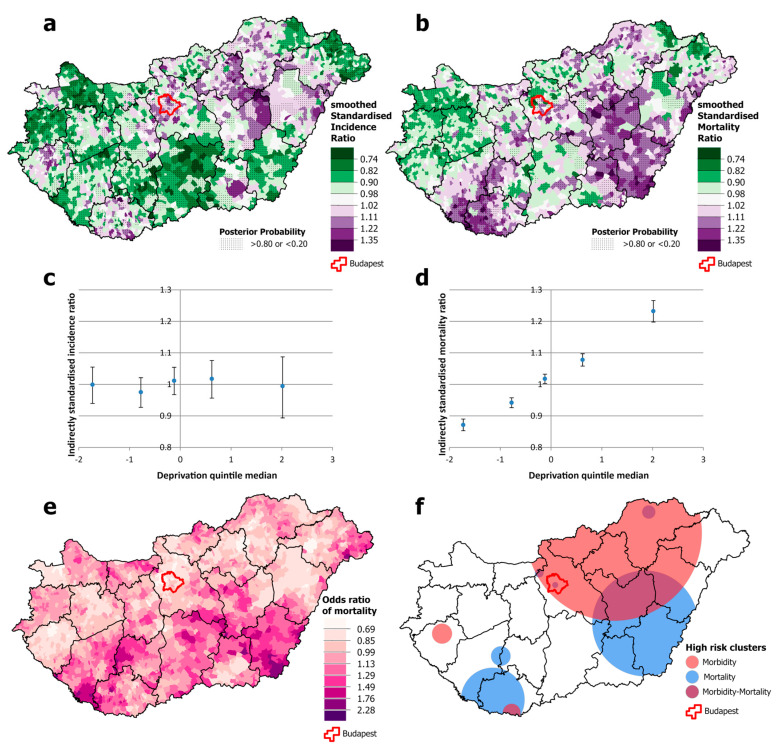
Spatial distribution of incidence (**a**) and mortality (**b**) at the municipality level; the relationship between deprivation and incidence (**c**) and mortality risk (**d**) by Deprivation Index quintile; odds ratio of mortality (**e**) and clusters of high incidence and mortality (**f**) due to malignant neoplasms, for females aged 25–64 years in Hungary, 2007–2018.

For females, the overlap between high-incidence and high-mortality areas is even smaller than that for men (Figure 4a,b). There is also a geographical discrepancy: high mortality occurs both in the south-western area and in an intense cluster in the south-eastern part of the country, including one county (Békés) where incidence is below the national average (Figure 4e,f). Clusters of high incidence and mortality are also observed in the Budapest area (Figure 4a,f). Although the spatial distribution of incidence was not associated with levels of deprivation, mortality increased significantly steeply from the least deprived to the most deprived quintile (Figure 4c,d). 

**Figure 5:**
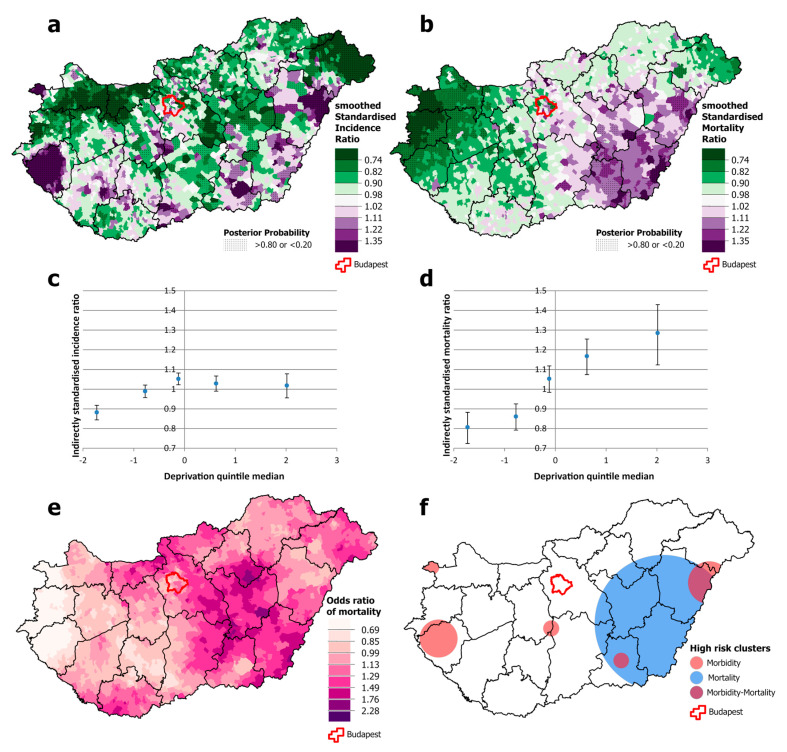
Spatial distribution of incidence (**a**) and mortality (**b**) at the municipality level; the relationship between deprivation and incidence (**c**) and mortality risk (**d**) by Deprivation Index quintile; odds ratio of mortality (**e**) and clusters of incidence and mortality (**f**) due to malignant neoplasms of the cervix uteri, for females aged 25–64 years in Hungary, 2007–2018.

There were clusters of significantly higher incidence of cervical cancer in one south-western county (Zala) and some counties in the south-eastern part of the country. However, premature mortality was only apparent in the south-eastern part of the country, and it was below the national average in the south-western counties (Figure 5a,b,e,f). It should be noted that the association between incidence and deprivation quintile is non-linear. It is below the national average only in the least deprived quintile, while in quintiles II-V, there was no association. However, there is more of a trend for mortality; although there was no difference between quintiles I and II, there was a significantly higher risk of death in all other quintiles (Figure 5c,d). 

**Figure 6:**
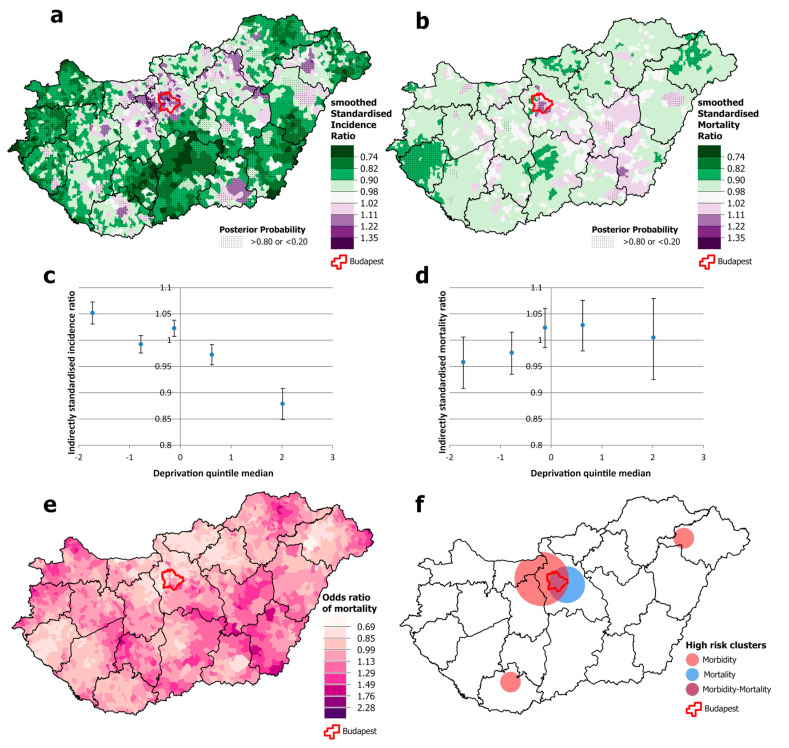
Spatial distribution of incidence (**a**) and mortality (**b**) at the municipality level; the relationship between deprivation and incidence (**c**) and mortality risk (**d**) by Deprivation Index quintile; odds ratio of mortality (**e**) and clusters of incidence and mortality (**f**) due to malignant neoplasms of the breast, for females aged 25–64 years in Hungary, 2007–2018.

The incidence of breast cancer was significantly higher throughout the capital, Budapest, and its surroundings than elsewhere. The same pattern was observed with premature mortality, in this case extending eastwards. There is also increased mortality in the south-eastern part of the country (Figure 6a,b,e,f). As noted earlier, this is the only cancer site included in this study for which there is a significant inverse association between deprivation and incidence; in quintile II, incidence is at the national average, while it is significantly higher in quintiles I and III and significantly lower in quintiles IV and V. The same trend cannot be observed for breast cancer mortality, where the trend is flat (Figure 6c,d).

**Figure 7:**
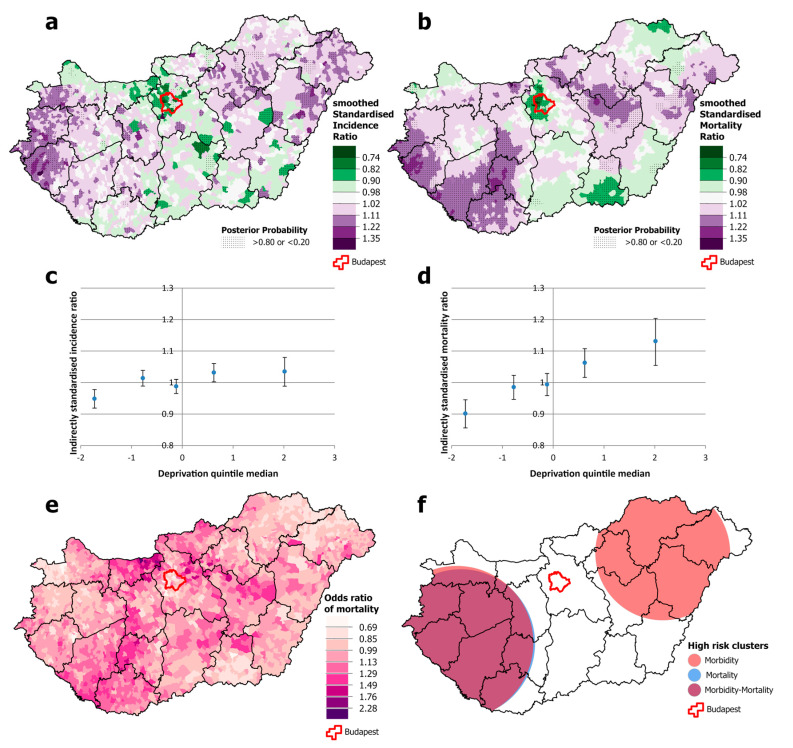
Spatial distribution of incidence (**a**) and mortality (**b**) at the municipality level; relationship between deprivation and relative incidence (**c**) and mortality risk (**d**) by Deprivation Index quintile; odds ratio of mortality (**e**) and clusters of relative incidence and mortality (**f**) due to malignant neoplasms of the colon, rectum, and anus for males aged 25–64 years, in Hungary, 2007–2018.

There is little variation in the incidence of colorectal neoplasia among males, with only the least deprived quintile having a lower-than-average incidence, while mortality increases with greater deprivation (Figure 7c,d). The risk of premature mortality is highest in the north-eastern and south-western parts of the country (Figure 7a,b,e,f). 

**Figure 8:**
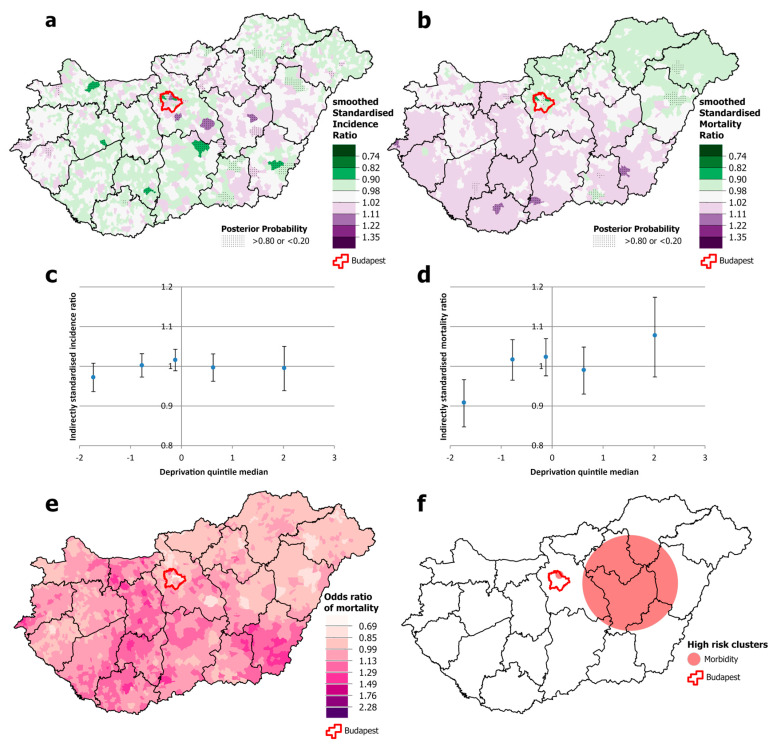
Spatial distribution of incidence (**a**) and mortality (**b**) at the municipality level; relationship between deprivation and incidence (**c**) and mortality risk (**d**) by Deprivation Index quintile; odds ratio of mortality (**e**) and clusters of incidence and mortality (**f**) due to malignant neoplasms of the colon, rectum, and anus, for females aged 25–64 years, in Hungary, 2007–2018.

Nor is there much variation in incidence for females (Figure 8a,c), but mortality is lower in the least deprived quintile (I) compared with the most deprived one (Figure 8d). It should be noted that incidence is higher, but mortality is lower than the national average in the capital (Figure 8a,b). The clusters of incidence and mortality do not fully overlap (Figure 8e,f). 

**Figure 9:**
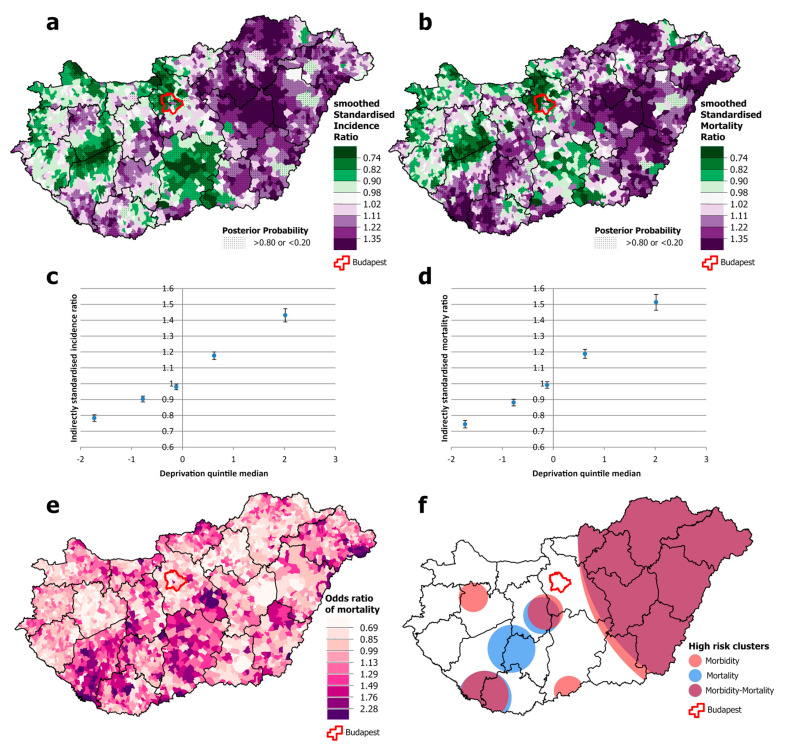
Spatial distribution of incidence (**a**) and mortality (**b**) at the municipality level; relationship between deprivation and relative incidence (**c**) and mortality risk (**d**) by Deprivation Index quintile; odds ratio of mortality (**e**) and clusters of relative incidence and mortality (**f**) due to malignant neoplasms of the trachea, bronchus, and lung, for males aged 25–64 years, in Hungary, 2007–2018.

There were statistically significant clusters of lung cancer incidence and mortality among males in eastern Hungary and on the south-western border and in the centre of the country, and these clusters overlap almost completely (Figure 9a,b,e,f). Both incidence and mortality are strongly associated with deprivation. Incidence is more than 20% lower than the national average in the least deprived quintile and more than 40% higher in the most deprived one, while the mortality gradient is even steeper, at around 25% lower in the least deprived quintile and more than 50% higher in the most deprived quintile (Figure 9c,d). However, there are clusters where incidence and mortality are misaligned (Figure 9e,f). 

**Figure 10:**
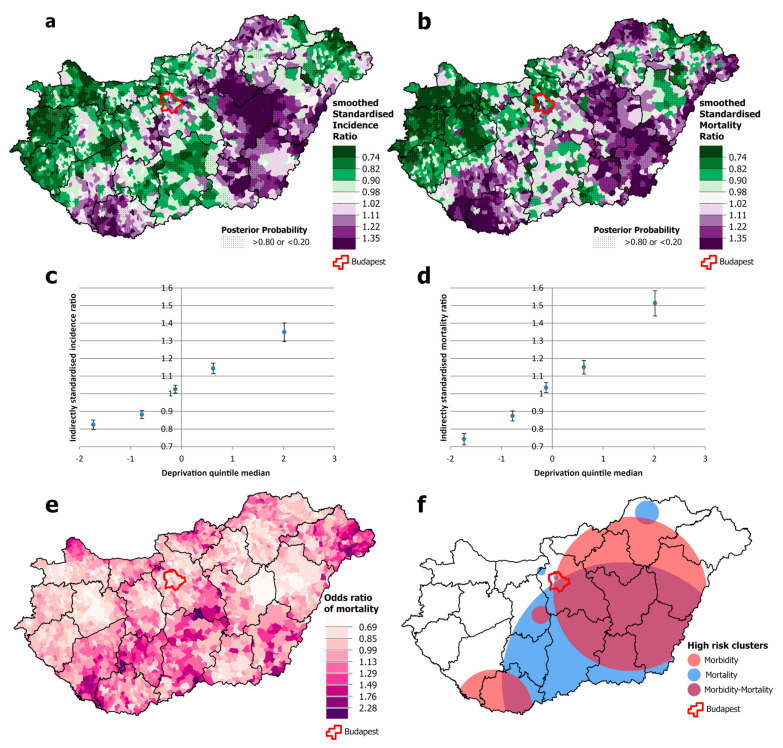
Spatial distribution of incidence (**a**) and mortality (**b**) at the municipality level; relationship between deprivation and incidence (**c**) and mortality risk (**d**) by Deprivation Index quintile; odds ratio of mortality (**e**) and clusters of incidence and mortality (**f**) due to malignant neoplasms of the trachea, bronchus, and lung, for females aged 25–64 years, in Hungary, 2007–2018.

For females, the spatial distributions of lung cancer incidence and mortality also overlapped (Figure 10a,b). As with males, both incidence and mortality are correlated with deprivation. Although the incidence is about 20% lower in the least deprived quintile and about 35% higher in the most deprived one (i.e., the gap is narrower), mortality shows a similarly large difference between the least and most deprived quintiles (25% lower vs. 50% higher risk) as for men (Figure 10c,d). Mortality clusters can be identified in areas where the incidence does not justify it (Figure 10e,f). 

Patterns of high incidence and mortality from neoplasms of the lip, oral cavity, and pharynx are similar for males, with clusters of both in the north-eastern and north-western parts of the country (Supplementary Figure S1a,b,f). For females, a significant cluster of increased incidence was observed only in the north-eastern part of the country (Supplementary Figure S2a,f), and while no other clusters were observed, some areas of higher mortality were found along the south-eastern border of the country (Supplementary Figure S2b). Although this clustering was not significant, the higher odds of mortality compared to incidence are clearly visible (Supplementary Figure S2e,f). Both incidence and mortality are strongly associated with deprivation in men (Supplementary Figure S1c,d), while for women, an increased incidence is only observed in quintiles IV and V, while mortality is significantly higher in quintile V compared to quintile I (Supplementary Figure S2c,d).

The incidence of pancreatic cancer was higher than average for both males and females in the north-eastern part of the country (Supplementary Figure S3a,f; Supplementary Figure S4a,f). A significant cluster of premature mortality was found only for males, which almost completely covers the cluster of high incidence (Supplementary Figure S3b,f; Figure S4b,f). A significant association with deprivation level was only observed for male mortality (Supplementary Figure S3d).

## 4. Discussion

There is a large body of research on the role of socioeconomic factors in cancer incidence and mortality [5,26,27]. To the best of our knowledge, our present study is the first to compare incidence and mortality from major cancers over the same period in an entire country. By using small geographical areas for comparison with each other and with a measure of deprivation, we could identify patterns of mortality that cannot be adequately explained by incidence or socioeconomic deprivation alone. Both incidence and mortality from all cancers overall show a significant positive association with deprivation, but, importantly, the slope of the increase in mortality with deprivation is much steeper than that for incidence. For the different cancer sites studied, mortality is always significantly higher in deprived areas, although the incidence of colorectal and pancreatic cancer in men and the incidence of colorectal cancer in women show no association with deprivation. As expected, breast cancer incidence is negatively associated with deprivation, but unexpectedly, mortality shows a significant positive association. It is important to note that both breast cancer incidence and mortality are significantly higher than the national average in the relatively non-deprived capital city of Budapest. 

Our disease mapping has identified three different patterns:

(i) The location of incidence and mortality clusters shows partial or almost complete overlap (e.g., all cancers for both sexes, colorectal cancer for males, lung cancer for both sexes in the country as a whole), but the odds of mortality vary widely compared to the national average mortality.
(ii) Mortality clusters are found in areas of relatively low incidence (in the south-west of the country for all cancers in both sexes and colorectal cancer in men).
(iii) Clusters of high incidence with mortality below the national average (cervical cancer in the western part of the country).

A more detailed examination of the different types of cancer points to areas for further study to understand the discrepancies between incidence and mortality in some places.

Previous studies have looked at cancer incidence or mortality, but very rarely both together, and especially not compared with each other. The most comprehensive European assessment of the extent, nature, and trends in inequalities in mortality by education, with data from 18 countries at multiple points over the period 1990–2015, concluded that “socioeconomic inequalities in mortality exist for most forms of cancer everywhere in Europe, with higher mortality rates for individuals at the lower ends of the social hierarchy” [5]. Other studies of geographical inequalities in mortality came from Germany [28] and from nine European urban areas [29]. A systematic review of 45 studies of cancer survival and socioeconomic conditions found marked inequalities that were, in part, associated with differences in treatment or comorbidities, although to different degrees [30]. A study of regional differences in England is an example of analyses of differences in incidence [31].

These patterns vary by cancer site. A comprehensive review of population-based epidemiological studies of socioeconomic inequalities in cancer incidence in Europe found that adults of lower socioeconomic status have an increased risk of head and neck, oesophago-gastric, liver and gallbladder, pancreatic, lung, kidney, bladder, penile, and cervical cancers, while those of higher socioeconomic status have an increased risk of thyroid, breast, prostate, and skin cancers, and the inequalities can be explained to varying degrees by known lifestyle factors, most notably smoking [32].

Importantly, associations between socioeconomic conditions and incidence or outcome are not inevitable. Cancer-specific, census-linked mortality data for the whole population by socioeconomic position were compared in 18 European countries for the period 1990–2015 in adults aged 40–79 [5]. Everywhere, less-educated individuals had higher mortality from almost all cancer types relative to their more educated counterparts, However, the magnitude of the inequalities varied greatly by country and over time. This was predominantly due to differences in cancer mortality among less educated groups; mortality from cancers in many sites was similar (and lower) among better-educated people in all countries. Inequalities were generally wider in the Baltic/Central/Eastern European countries and narrower in Southern Europe, although large and increasing inequalities were found among women in Northern Europe (relative risk of all cancer mortality for lower- versus higher-educated ≥1.4 in Denmark, Norway, Sweden, Finland, and England/Wales). For men, the absolute differences in rates (per 100,000 person-years) of total cancer mortality between less- and better-educated groups ranged from 110 (Sweden) to 559 (Czech Republic), and for women, from approximately zero (Slovenia, Italy, Spain) to 176 (Denmark).

However, few studies have examined both incidence and mortality together. This is probably partly because, as noted above, ideally one would look at both incidence and survival, but, in practice, this is not yet possible in many settings. We argue that, while acknowledging the limitations, analyses that compare patterns of incidence and mortality can provide a better understanding than those that look at the two separately. 

It is important to note that in addition to the strengths of our study described above, it has several limitations. The data used in our study are not cancer survival data from individual records. These data are currently not available in Hungary, as they are in most countries. In addition, the mortality and incidence data used were not individually linked. Therefore, our analysis of mortality and incidence was performed for the largest possible time interval. Nevertheless, it is also a limitation that deaths that occurred during this period may not have been diagnosed with the disease during this period, or there may be patients who were diagnosed with the disease but died after this period, and the data are also influenced by the different survival times for each type of tumour. Although the quality of mortality and incidence data is improving, some uncertainties remain due to different coding practices for the primary cause of death and/or incidence.

As is well known, ecological studies have a number of limitations. Associations found in research do not prove causality. For example, deprivation associated with mortality at the population level may not necessarily be associated with disease at the individual level. However, hierarchical Bayesian methodology allows spatial data to be analysed and mapped and their relationships to be analysed at the highest possible level of resolution.

The limited timeliness of the DI is another limitation of this study. Most of the indicators at the municipal level are only available from the most recent census (2011), and therefore, as with all such indices, the indicators may become less appropriate over time. A new census may provide information for updating the required indicators. However, it should be noted that the spatial distribution of deprived areas has not changed significantly, with the same patterns of underdevelopment identified 40 years ago [11]. One limitation is that the analysis is not based on the most recent data, but on data for the 2009–2018 decade. This is because Hungarian data on incidence by municipality have not been updated since the pandemic. The COVID-19 pandemic has led to an unprecedented reversal in adult mortality at global, regional, and national levels, and both cancer incidence and mortality have been severely affected [30], with delays in diagnosis, disruptions in cancer screening programmes, and interruptions in planned therapies [33].

For all cancers combined and for individual cancer sites, detailed evaluation helps to identify discrepancies between incidence and mortality at the local level. It is beyond the scope of this study to determine the reasons for these discrepancies, but by publishing these findings, we hope that those responsible for public health policy will use them as a basis for more detailed explorations.

We also believe that this information can be used to inform the implementation of health policies. For example, in 2018, a Mobile Health Screening Programme was launched in Hungary, offering a portfolio of screening activities, including for cervical, breast, and oral cancers and melanoma. Mobile screening units were used to target socioeconomically disadvantaged rural settlements (reaching about 7000 people in 142 settlements). However, this process took no account of information on the incidence and mortality of the cancers being screened. As our analysis shows, the fact that an area is deprived is not in itself sufficient to identify a population at increased risk (the distribution of deaths from cervical cancer does not match with the distribution of deprivation, and this is even less true for breast cancer).

Similarly, knowing the location of areas with high mortality but relatively low incidence may point to a need for understanding why this anomaly exists, such as a problem with screening or treatment systems. In contrast, areas with high incidence but below-average mortality may provide examples of best practices for public health services (which of course does not mean that the underlying causes of high incidence should be ignored).

More broadly, there is a clear need to improve the socioeconomic situation, which will benefit the public health situation in many ways (including cancer incidence and mortality) [26]. In their ecological study, Lortet-Tieulent et al. [27] assessed the association between national socioeconomic level and incidence and mortality for all cancers combined and 27 cancer types in 175 countries for 2018, using estimates from GLOBOCAN. They related their findings to the Sustainable Development Index (SDI) used by the United Nations Development Programme. Cancer incidence was strongly and positively associated with the SDI for all cancers combined and for many cancer sites, in both sexes. Conversely, the relationship between the SDI and cancer mortality was less clear. However, this improvement—even with strong government support—can only be achieved in the long term, but action is needed now.

Reducing deprivation is an important objective, but one that takes time. For now, targeted interventions are needed without delay for populations in areas of particularly high mortality, as identified by spatial data analysis.

## 5. Conclusions

In recognition of the significant influence of socioeconomic conditions on both cancer incidence and mortality and the importance of reducing inequalities, policy responses require information on the geographical distribution of incidence and mortality. Explaining cancer incidence and its mortality burden solely in terms of socioeconomic conditions (deprivation levels) and related lifestyle factors is too simplistic. Geospatial analysis can help to identify areas with discrepancies in cancer incidence and mortality, and after further exploration, appropriate preventive, and/or healthcare interventions can be developed and implemented.

## Figures and Tables

**Table 1:** The association between deprivation and incidence in the Hungarian population aged 25–64 years due to all and selected malignant neoplasms in Hungary, 2007–2018.

	Male	Female
	RR [95% CI]	
Malignant neoplasms	1.02 [1.02–1.03]	1.02 [1.01–1.03]
Malignant neoplasms of lip, oral cavity, and pharynx	1.10 [1.08–1.13]	1.08 [1.04–1.12]
Malignant neoplasm of colon, rectum, and anus	1.00 [0.99–1.02]	1.01 [0.99–1.03]
Malignant neoplasm of pancreas	1.01 [0.98–1.04]	1.05 [1.01–1.08]
Malignant neoplasm of trachea, bronchus, and lung	1.14 [1.12–1.16]	1.20 [1.17–1.22]
Malignant neoplasm of breast	..	0.98 [0.96–0.99]
Malignant neoplasm of cervix uteri	..	1.05 [1.02–1.08]

RR: relative risk, the increase in the risk of incidence for each unit increase in the Deprivation Index. CI: credible interval. Numbers in red: RR has a significantly positive association; numbers in blue: RR has a significantly reverse association; numbers in black: RR has no significant association.

**Table 2:** The association between deprivation and mortality in the Hungarian population aged 25–64 years due to all and selected malignant neoplasms in Hungary, 2007–2018.

	Male	Female
	RR [95% CI]	
Malignant neoplasms	1.11 [1.10–1.13]	1.13 [1.11–1.14]
Malignant neoplasms of lip, oral cavity, and pharynx	1.14 [1.10–1.17]	1.08 [1.02–1.15]
Malignant neoplasm of colon, rectum, and anus	1.05 [1.03–1.08]	1.04 [1.01–1.07]
Malignant neoplasm of pancreas	1.05 [1.02–1.08]	1.04 [1.01–1.08]
Malignant neoplasm of trachea, bronchus, and lung	1.18 [1.16–1.20]	1.27 [1.24–1.30]
Malignant neoplasm of breast	..	1.04 [1.01–1.07]
Malignant neoplasm of cervix uteri	..	1.16 [1.11–1.21]

RR: relative risk, the increase in the risk of mortality for each unit increase in the Deprivation Index. CI: credible interval, numbers in red: RR has a significantly positive association.

## Data Availability

The data presented in this study are available on request from the corresponding author. The data are not publicly available due to privacy or ethical restrictions.

## Supplementary Materials

The following supporting information can be downloaded at https://www.mdpi.com/article/10.3390/cancers16162917/s1: Supplementary Table S1: Relationship between deprivation and relative incidence and mortality risks due to all malignant neoplasms for the ages of 25–64, by Deprivation Index (DI) quintile in Hungary, 2007–2018. Supplementary Table S2: Relationship between deprivation and relative incidence and mortality risks due to lung cancer for the ages of 25–64, by Deprivation Index (DI) quintile in Hungary, 2007–2018. Supplementary Table S3: Relationship between deprivation and relative incidence and mortality risks due to colorectal cancer for the ages of 25–64, by Deprivation Index (DI) quintile in Hungary, 2007–2018. Supplementary Table S4: Relationship between deprivation and relative incidence and mortality risks due to breast and cervical cancer for the ages of 25–64, by Deprivation Index (DI) quintile in Hungary, 2007–2018. Supplementary Table S5: Relationship between deprivation and relative incidence and mortality risks due to neoplasms of the lip, oral cavity, and pharynx for the ages of 25–64, by Deprivation Index (DI) quintile in Hungary, 2007–2018. Supplementary Table S6: Relationship between deprivation and relative incidence and mortality risks due to pancreas neoplasms for the ages of 25–64, by Deprivation Index (DI) quintile in Hungary, 2007–2018. Supplementary Table S7: Results of chi-square tests for homogeneity and linear trends for testing association in risk analysis. Supplementary Figure S1: Spatial distribution of incidence (a) and mortality (b); relationship between deprivation and relative incidence (c) and mortality risk (d) by Deprivation Index quintile; odds ratio of mortality (e) and clusters of relative incidence and mortality (f) due to all malignant neoplasms of the lip, oral cavity, and pharynx, aged 25–64 years, for males in Hungary, at the municipality level, 2007–2018. Supplementary Figure S2: Spatial distribution of incidence (a) and mortality (b); relationship between deprivation and relative incidence (c) and mortality risk (d) by Deprivation Index quintile; odds ratio of mortality (e) and clusters of relative incidence and mortality (f) due to all malignant neoplasms of the lip, oral cavity, and pharynx, aged 25–64 years, for females in Hungary, at the municipality level, 2007–2018. Supplementary Figure S3: Spatial distribution of incidence (a) and mortality (b); relationship between deprivation and relative incidence (c) and mortality risk (d) by Deprivation Index quintile; odds ratio of mortality (e) and clusters of relative incidence and mortality (f) due to all malignant neoplasms of the pancreas, aged 25–64 years, for males in Hungary, at the municipality level, 2007–2018. Supplementary Figure S4: Spatial distribution of incidence (a) and mortality (b); relationship between deprivation and relative incidence (c) and mortality risk (d) by Deprivation Index quintile; odds ratio of mortality (e) and clusters of relative incidence and mortality (f) due to all malignant neoplasms of the pancreas, aged 25–64 years, for females in Hungary, at the municipality level, 2007–2018.
