# Correction: Ádány et al. Discrepancies between the Spatial Distribution of Cancer Incidence and Mortality as an Indicator of Unmet Needs in Cancer Prevention and/or Treatment in Hungary. *Cancers* 2024, *16*, 2917

**DOI:** 10.3390/cancers16233964

**Published:** 2024-11-26

**Authors:** Róza Ádány, Attila Juhász, Csilla Nagy, Bernadett Burkali, Péter Pikó, Martin McKee, Beatrix Oroszi

**Affiliations:** 1 Epidemiology and Surveillance Centre, Semmelweis University, 25. Üllői Street, 1085 Budapest, Hungary; juhasz.attila@semmelweis.hu (A.J.); nagy.csilla@semmelweis.hu (C.N.); burkali.bernadett@semmelweis.hu (B.B.); piko.peter@semmelweis.hu (P.P.); oroszi.beatrix@semmelweis.hu (B.O.); 2 Department of Public Health and Epidemiology, Faculty of Medicine, University of Debrecen, 26. Kassai Street, 4028 Debrecen, Hungary; 3 HUN-REN Public Health Research Group, University of Debrecen, 26. Kassai Street, 4028 Debrecen, Hungary; 4 Department of Preventive Medicine and Public Health, Semmelweis University, 1085 Budapest, Hungary; 5 Department of Quality Management in Healthcare and Infection Control, Petz Aladár Teaching Hospital of Győr-Moson-Sopron County, 2-4. Vasvári Pál Street, 9024 Győr, Hungary; 6 Department of Health Services Research and Policy, London School of Hygiene and Tropical Medicine, Keppel Street, London WC1E 7HT, UK; martin.mckee@lshtm.ac.uk

## 1. Error in Figure

In the original publication [1], there was a mistake in Figure 1 as published. “The number of cases in Figure 1 is incorrect”. The corrected Figure 1 appears below. 

## 2. Text Correction

There was an error in the original publication. The case numbers in the description of the results presented in Figure 1 are incorrect.

A correction has been made to “*3.1. Incidence and Mortality of Malignant Neoplasms*”:

In the Hungarian population aged 25–64, there were about half a million incident cases (males: 230,771; females: 242,726) of malignant neoplasms between 2007 and 2018, with 85,968 deaths in males and 57,094 deaths in females.

The incidence of malignant neoplasms (excluding C44) showed a modestly increasing trend for females and a slightly decreasing trend for males between 2007 and 2018. Mortality from malignant neoplasms decreased for both sexes, with a steeper decrease for males over the period studied.

The authors apologize for any inconvenience caused and state that the scientific conclusions are unaffected. This correction was approved by the Academic Editor. The original publication has also been updated.

## Figures and Tables

**Figure 1:**
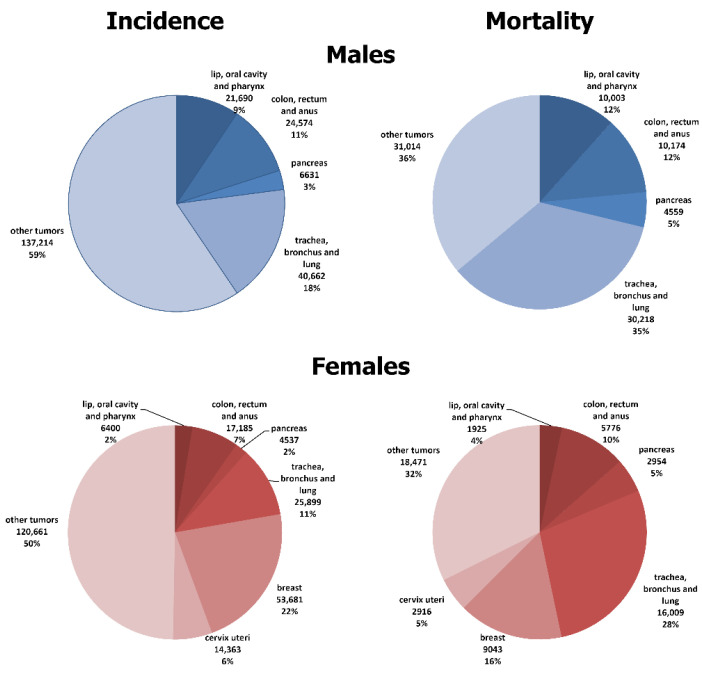
Proportion of incidence and premature mortality due to selected major malignant neoplasms in the Hungarian population, at ages 25–64, 2007–2018.
