# Supplementary material for: Discrepancies between the Spatial Distribution of Cancer Incidence and Mortality as an Indicator of Unmet Needs in Cancer Prevention and/or Treatment in Hungary
Source: Cancers (Basel). 2024 Aug 22;16(16):2917. doi: 10.3390/cancers16162917 (PMC11353249; doi:10.3390/cancers16162917)
Supplement: Supplementary file 1 [file cancers-16-02917-s001.zip › cancers-3116887-supplementary.pdf]

# Supplementary appendix

## Table of contents

**Supplementary Table S1**: Relationship between the deprivation and relative incidence, mortality due to all malignant neoplasms in aged of 25-64, by Deprivation Index (DI) quintile in Hungary, 2007-2018................. 2

**Supplementary Table S2**: Relationship between the deprivation and relative incidence, mortality due to lung cancer in aged of 25-64, by Deprivation Index (DI) quintile in Hungary, 2007-2018........................................... 2

**Supplementary Table S3**: Relationship between the deprivation and relative incidence, mortality due to colorectal cancer in aged of 25-64, by Deprivation Index (DI) quintile in Hungary, 2007-2018......................... 2

**Supplementary Table S4**: Relationship between the deprivation and relative incidence, mortality due to breast and cervical cancer in aged of 25-64, by Deprivation Index (DI) quintile in Hungary, 2007-2018.......... 3

**Supplementary Table S5**: Relationship between the deprivation and relative incidence, mortality due to neoplasms of the lip, oral cavity and pharynx in aged of 25-64, by Deprivation Index (DI) quintile in Hungary, 2007-2018........................................................................................................................................ 3

**Supplementary Table S6**: Relationship between the deprivation and relative incidence, mortality due to pancreas neoplasms in aged of 25-64, by Deprivation Index (DI) quintile in Hungary, 2007-2018................... 3

**Supplementary Table S7**: Results of Chi-square tests for homogeneity and linear trends to test association in risk analysis ........................................................................................................................................ 4

**Supplementary Figure S1**: Spatial distribution of incidence (a) and mortality (b); relationship between the deprivation and relative incidence (c) and mortality risk (d) by Deprivation Index quintile; odds ratio of mortality (e) and clusters of relative incidence, mortality (f) due to all malignant neoplasms of lip, oral cavity and pharynx, aged 25-64 years, for males In Hungary, at municipality level, 2007-2018.................................. 5

**Supplementary Figure S2**: Spatial distribution of incidence (a) and mortality (b); relationship between the deprivation and relative incidence (c) and mortality risk (d) by Deprivation Index quintile; odds ratio of mortality (e) and clusters of relative incidence, mortality (f) due to all malignant neoplasms of lip, oral cavity and pharynx, aged 25-64 years, for females In Hungary, at municipality level, 2007-2018................................ 6

**Supplementary Figure S3**: Spatial distribution of incidence (a) and mortality (b); relationship between the deprivation and relative incidence (c) and mortality risk (d) by Deprivation Index quintile; odds ratio of mortality (e) and clusters of relative incidence, mortality (f) due to all malignant neoplasms of pancreas, aged 25-64 years, for males In Hungary, at municipality level, 2007-2018..................................................................... 7

**Supplementary Figure S4**: Spatial distribution of incidence (a) and mortality (b); relationship between the deprivation and relative incidence (c) and mortality risk (d) by Deprivation Index quintile; odds ratio of mortality (e) and clusters of relative incidence, mortality (f) due to all malignant neoplasms of pancreas, aged 25-64 years, for females In Hungary, at municipality level, 2007-2018.................................................................. 8

**Supplementary Table S1**: Relationship between the deprivation and relative incidence, mortality due to all malignant neoplasms in aged of 25-64, by Deprivation Index (DI) quintile in Hungary, 2007-2018.

| **DI quintiles** (range of DI values) | **Males** | **Females** | **Males** | **Females** |
|---|---|---|---|---|
| | **Indirectly standardized incidence ratio [95%CI] (number of cases)** | | **Indirectly standardized mortality ratio [95%CI] (number of cases)** | |
| **I. (least deprived)** (-4.90 <= DI <= -1.13) | 0.94 [0.93 - 0.95] (33,096) | 0.99 [0.98 - 1.00] (36,001) | 0.81 [0.80 - 0.83] (11,996) | 0.87 [0.85 - 0.89] (8492) |
| **II.** (-1.13 < DI <= -0.43) | 0.96 [0.95 - 0.97] (50,585) | 0.98 [0.97 - 0.99] (53,521) | 0.93 [0.92 - 0.94] (20,402) | 0.94 [0.92 - 0.96] (13,901) |
| **III.** (-0.43 < DI <= 0.22) | 0.98 [0.97 - 0.99] (61,866) | 1.00 [0.99 - 1.01] (66,303) | 0.98 [0.97 - 0.99] (25,808) | 1.02 [1.00 - 1.03] (18,044) |
| **IV.** (0.22 < DI <= 1.06) | 1.06 [1.05 - 1.07] (42,081) | 1.02 [1.01 - 1.03] (40,940) | 1.13 [1.12 - 1.15] (18,721) | 1.08 [1.06 - 1.10] (11,624) |
| **V. (most deprived)** (1.06 < DI <= 8.00) | 1.15 [1.14 - 1.17] (18,565) | 1.02 [1.00 - 1.04] (15,621) | 1.35 [1.32 - 1.38] (9041) | 1.23 [1.20 - 1.27] (5033) |

DI: Deprivation Index
CI: Confidence Intervals

**Supplementary Table S2**: Relationship between the deprivation and relative incidence, mortality due to lung cancer in aged of 25-64, by Deprivation Index (DI) quintile in Hungary, 2007-2018.

| **DI quintiles** (range of DI values) | **Males** | **Females** | **Males** | **Females** |
|---|---|---|---|---|
| | **Indirectly standardized incidence ratio [95%CI] (number of cases)** | | **Indirectly standardized mortality ratio [95%CI] (number of cases)** | |
| **I. (least deprived)** (-4.90 <= DI <= -1.13) | 0.78 [0.76 - 0.80] (5460) | 0.82 [0.79 - 0.85] (3638) | 0.74 [0.72 - 0.76] (3860) | 0.74 [0.71 - 0.77] (2026) |
| **II.** (-1.13 < DI <= -0.43) | 0.90 [0.88 - 0.92] (9355) | 0.88 [0.86 - 0.90] (5903) | 0.88 [0.86 - 0.90] (6783) | 0.87 [0.84 - 0.90] (3619) |
| **III.** (-0.43 < DI <= 0.22) | 0.98 [0.96 - 0.99] (12,142) | 1.02 [1.00 - 1.04] (8256) | 0.99 [0.97 - 1.01] (9135) | 1.03 [1.00 - 1.06] (5149) |
| **IV.** (0.22 < DI <= 1.06) | 1.17 [1.15 - 1.20] (9188) | 1.14 [1.11 - 1.17] (5601) | 1.18 [1.16 - 1.21] (6896) | 1.15 [1.11 - 1.19] (3486) |
| **V. (most deprived)** (1.06 < DI <= 8.00) | 1.43 [1.39 - 1.47] (4517) | 1.35 [1.29 - 1.40] (2501) | 1.51 [1.46 - 1.56] (3544) | 1.51 [1.44 - 1.58] (1729) |

DI: Deprivation Index
CI: Confidence Intervals

**Supplementary Table S3**: Relationship between the deprivation and relative incidence, mortality due to colorectal cancer in aged of 25-64, by Deprivation Index (DI) quintile in Hungary, 2007-2018.

| **DI quintiles** (range of DI values) | **Males** | **Females** | **Males** | **Females** |
|---|---|---|---|---|
| | **Indirectly standardized incidence ratio [95%CI] (number of cases)** | | **Indirectly standardized mortality ratio [95%CI] (number of cases)** | |
| **I. (least deprived)** (-4.90 <= DI <= -1.13) | 0.94 [0.92 - 0.97] (4002) | 0.97 [0.93 - 1.00] (2856) | 0.90 [0.85 - 0.94] (1577) | 0.90 [0.85 - 0.97] (897) |
| **II.** (-1.13 < DI <= -0.43) | 1.01 [0.98 - 1.03] (6348) | 1.00 [0.97 - 1.03] (4454) | 0.98 [0.94 - 1.02] (2556) | 1.01 [0.96 - 1.07] (1520) |
| **III.** (-0.43 < DI <= 0.22) | 0.98 [0.96 - 1.01] (7397) | 1.01 [0.98 - 1.04] (5420) | 0.99 [0.96 - 1.03] (3082) | 1.02 [0.97 - 1.07] (1836) |
| **IV.** (0.22 < DI <= 1.06) | 1.03 [1.00 - 1.06] (4861) | 0.99 [0.96 - 1.03] (3233) | 1.06 [1.01 - 1.11] (2072) | 0.99 [0.93 - 1.05] (1080) |
| **V. (most deprived)** (1.06 < DI <= 8.00) | 1.03 [0.99 - 1.08] (1966) | 0.99 [0.94 - 1.05] (1222) | 1.13 [1.06 - 1.20] (887) | 1.07 [0.98 - 1.18] (443) |

DI: Deprivation Index
CI: Confidence Intervals

**Supplementary Table S4**: Relationship between the deprivation and relative incidence, mortality due to breast and cervical cancer in aged of 25-64, by Deprivation Index (DI) quintile in Hungary, 2007-2018.

| **DI quintiles** (range of DI values) | **Breast cancer** | **Cervical cancer** | **Breast cancer** | **Cervical cancer** |
|---|---|---|---|---|
| | **Indirectly standardized incidence ratio [95%CI] (number of cases)** | | **Indirectly standardized mortality ratio [95%CI] (number of cases)** | |
| **I. (least deprived)** (-4.90 <= DI <= -1.13) | 1.05 [1.03 - 1.07] (9654) | 0.82 [0.84 - 0.92] (2177) | 0.95 [0.91 - 1.00] (1480) | 0.80 [0.73 - 0.89] (402) |
| **II.** (-1.13 < DI <= -0.43) | 0.99 [0.97 - 1.00] (13,746) | 0.98 [0.95 - 1.02] (3660) | 0.97 [0.93 - 1.01] (2280) | 0.86 [0.79 - 0.93] (647) |
| **III.** (-0.43 < DI <= 0.22) | 1.02 [1.00 - 1.03] (17,028) | 1.05 [1.02 - 1.08] (4687) | 1.02 [0.98 - 1.06] (2874) | 1.05 [0.98 - 1.12] (952) |
| **IV.** (0.22 < DI <= 1.06) | 0.97 [0.95 - 0.99] (9841) | 1.02 [0.99 - 1.06] (2766) | 1.02 [0.98 - 1.07] (1756) | 1.16 [1.08 - 1.26] (642) |
| **V. (most deprived)** (1.06 < DI <= 8.00) | 0.87 [0.85 - 0.90] (3412) | 1.01 [0.9 - 1.08] (1073) | 1.00 [0.93 - 1.08] (653) | 1.28 [1.14 - 1.44] (273) |

DI: Deprivation Index
CI: Confidence Intervals

**Supplementary Table S5**: Relationship between the deprivation and relative incidence, mortality due to neoplasms of the lip, oral cavity and pharynx in aged of 25-64, by Deprivation Index (DI) quintile in Hungary, 2007-2018.

| **DI quintiles** (range of DI values) | **Males** | **Females** | **Males** | **Females** |
|---|---|---|---|---|
| | **Indirectly standardized incidence ratio [95%CI] (number of cases)** | | **Indirectly standardized mortality ratio [95%CI] (number of cases)** | |
| **I. (least deprived)** (-4.90 <= DI <= -1.13) | 0.80 [0.77 - 0.83] (2974) | 0.94 [0.89 - 1.00] (1026) | 0.73 [0.69 - 0.77] (1242) | 0.87 [0.77 - 0.98] (286) |
| **II.** (-1.13 < DI <= -0.43) | 0.91 [0.89 - 0.94] (5056) | 0.95 [0.91 - 1.00] (1580) | 0.93 [0.89 - 0.97] (2365) | 0.97 [0.88 - 1.06] (482) |
| **III.** (-0.43 < DI <= 0.22) | 0.97 [0.95 - 0.99] (6441) | 0.96 [0.92 - 1.01] (1919) | 0.99 [0.95 - 1.02] (3020) | 0.99 [0.91 - 1.07] (594) |
| **IV.** (0.22 < DI <= 1.06) | 1.13 [1.10 - 1.16] (4743) | 1.12 [1.06 - 1.18] (1358) | 1.15 [1.10 - 1.20] (2230) | 1.08 [0.98 - 1.20] (397) |
| **V. (most deprived)** (1.06 < DI <= 8.00) | 1.44 [1.39 - 1.50] (2476) | 1.12 [1.02 - 1.22] (517) | 1.44 [1.36 - 1.53] (1146) | 1.20 [1.03 - 1.40] (166) |

DI: Deprivation Index
CI: Confidence Intervals

**Supplementary Table S6**: Relationship between the deprivation and relative incidence, mortality due to pancreas neoplasms in aged of 25-64, by Deprivation Index (DI) quintile in Hungary, 2007-2018.

| **DI quintiles** (range of DI values) | **Males** | **Females** | **Males** | **Females** |
|---|---|---|---|---|
| | **Indirectly standardized incidence ratio [95%CI] (number of cases)** | | **Indirectly standardized mortality ratio [95%CI] (number of cases)** | |
| **I. (least deprived)** (-4.90 <= DI <= -1.13) | 0.90 [0.84 - 0.97] (1097) | 0.96 [0.88 - 1.05] (792) | 0.96 [0.91 - 1.02] (710) | 1.02 [0.95 - 1.09] (487) |
| **II.** (-1.13 < DI <= -0.43) | 1.00 [0.95 - 1.06] (1729) | 0.94 [0.88 - 1.02] (1119) | 1.02 [0.97 - 1.07] (1171) | 0.95 [0.89 - 1.01] (725) |
| **III.** (-0.43 < DI <= 0.22) | 0.95 [0.90 - 1.00] (1906) | 1.02 [0.96 - 1.09] (1400) | 0.94 [0.90 - 0.98] (1325) | 0.99 [0.94 - 1.04] (944) |
| **IV.** (0.22 < DI <= 1.06) | 1.07 [1.00 - 1.14] (1342) | 1.03 [0.95 - 1.12] (864) | 1.05 [0.99 - 1.11] (937) | 1.00 [0.94 - 1.07] (577) |
| **V. (most deprived)** (1.06 < DI <= 8.00) | 1.17 [1.06 - 1.29] (557) | 1.05 [0.92 - 1.20] (362) | 1.08 [0.99 - 1.17] (416) | 1.12 [1.01 - 1.24] (221) |

DI: Deprivation Index
CI: Confidence Intervals

**Supplementary Table S7**: Results of Chi-square tests for homogeneity and linear trends to test association in risk analysis

| **Males** | **Incidence** | | **Mortality** | |
|---|---|---|---|---|
| | $\chi_{homogeneity}$ | $P_{homogeneity}$ | $\chi_{linearity}$ | $P_{linearity}$ |
| **Malignant neoplasms of lip, oral cavity and pharynx** | 595.27 | 0.00 | 339.92 | 0.00 |
| **Malignant neoplasms** | 760.80 | 0.00 | 1739.64 | 0.00 |
| **Malignant neoplasm of colon, rectum and anus** | 20.55 | 0.00 | 38.82 | 0.00 |
| **Malignant neoplasm of pancreas** | 15.78 | 0.01 | 25.06 | 0.00 |
| **Malignant neoplasm of trachea, bronchus and lung** | 1259.09 | 0.00 | 1269.85 | 0.00 |
| **Females** | **Incidence** | | **Mortality** | |
| | $\chi_{homogeneity}$ | $P_{homogeneity}$ | $\chi_{linearity}$ | $P_{linearity}$ |
| **Malignant neoplasms of lip, oral cavity and pharynx** | 34.12 | 0.00 | 14.17 | 0.01 |
| **Malignant neoplasms** | 62.17 | 0.00 | 500.36 | 0.00 |
| **Malignant neoplasm of colon, rectum and anus** | 98.53 | 0.00 | 7.04 | 0.13 |
| **Malignant neoplasm of pancreas** | 3.67 | 0.45 | 12.29 | 0.01 |
| **Malignant neoplasm of trachea, bronchus and lung** | 7.62 | 0.11 | 4.67 | 0.32 |
| **Malignant neoplasm of breast** | 98.53 | 0.00 | 7.04 | 0.13 |
| **Malignant neoplasm of cervix uteri** | 3.67 | 0.45 | 12.29 | 0.02 |

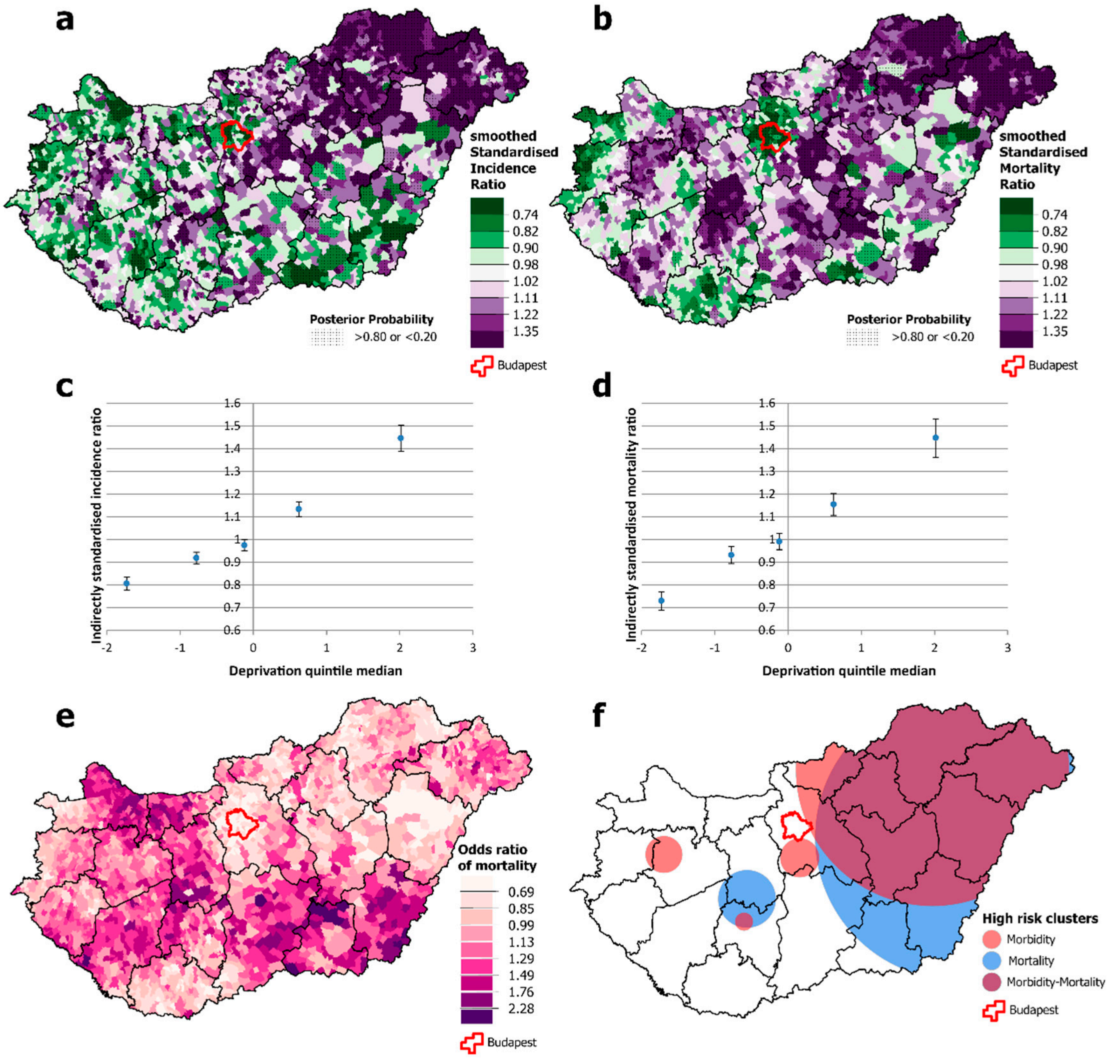


**Supplementary Figure S1**: Spatial distribution of incidence (a) and mortality (b); relationship between the deprivation and relative incidence (c) and mortality risk (d) by Deprivation Index quintile; odds ratio of mortality (e) and clusters of relative incidence, mortality (f) due to all malignant neoplasms of lip, oral cavity and pharynx, aged 25-64 years, for males In Hungary, at municipality level, 2007-2018.

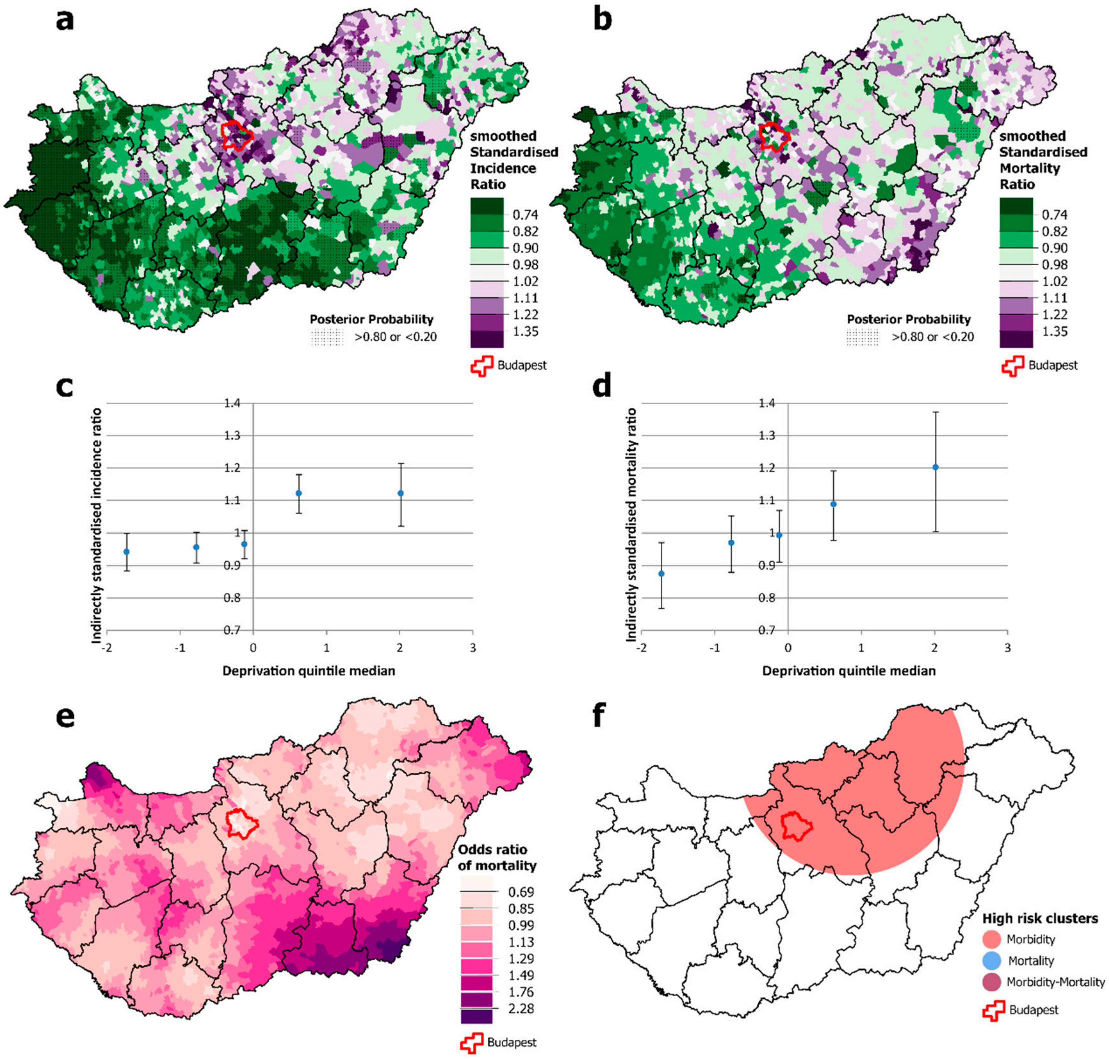


**Supplementary Figure S2**: Spatial distribution of incidence (a) and mortality (b); relationship between the deprivation and relative incidence (c) and mortality risk (d) by Deprivation Index quintile; odds ratio of mortality (e) and clusters of relative incidence, mortality (f) due to all malignant neoplasms of lip, oral cavity and pharynx, aged 25-64 years, for females In Hungary, at municipality level, 2007-2018.

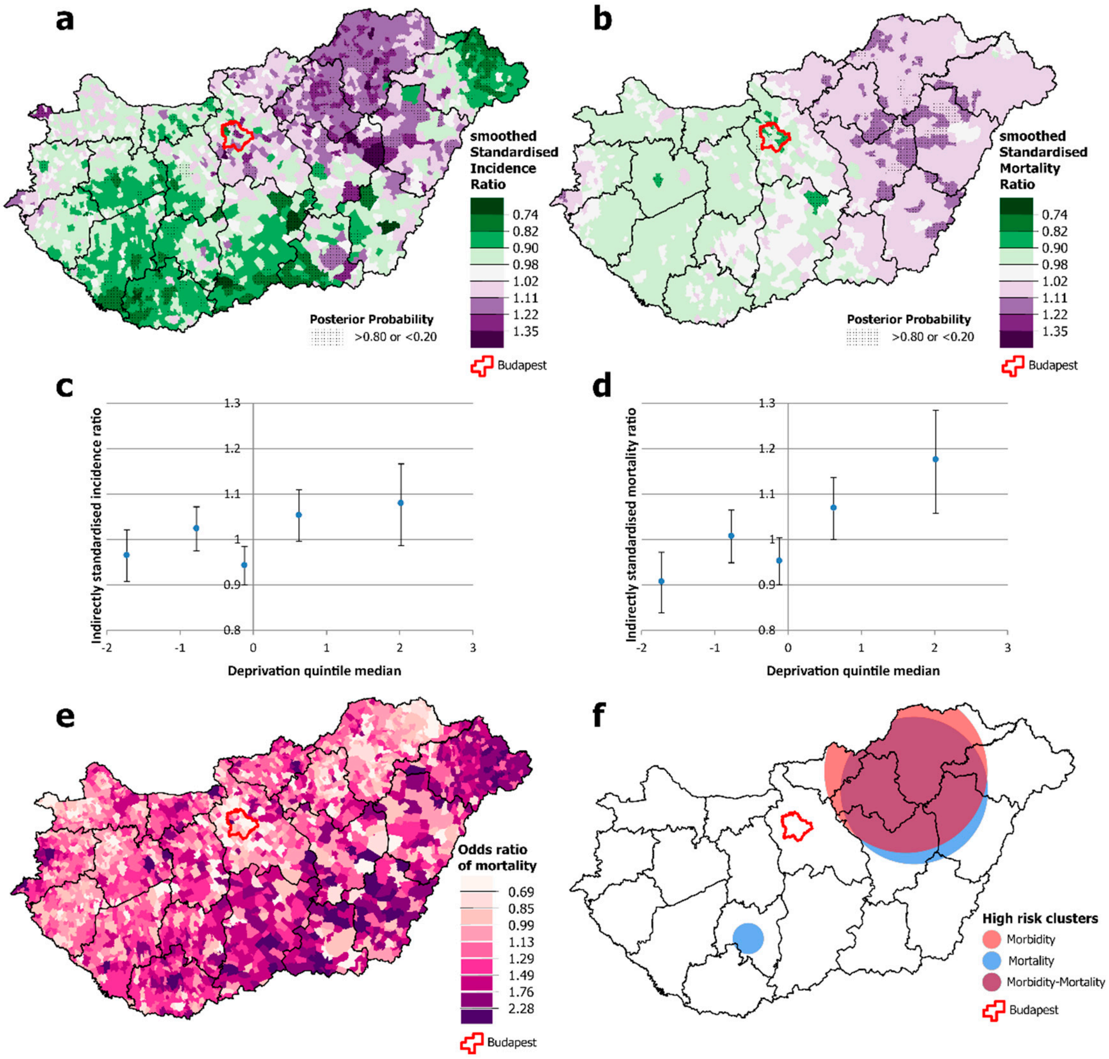


**Supplementary Figure S3**: Spatial distribution of incidence (a) and mortality (b); relationship between the deprivation and relative incidence (c) and mortality risk (d) by Deprivation Index quintile; odds ratio of mortality (e) and clusters of relative incidence, mortality (f) due to all malignant neoplasms of pancreas, aged 25-64 years, for males In Hungary, at municipality level, 2007-2018.

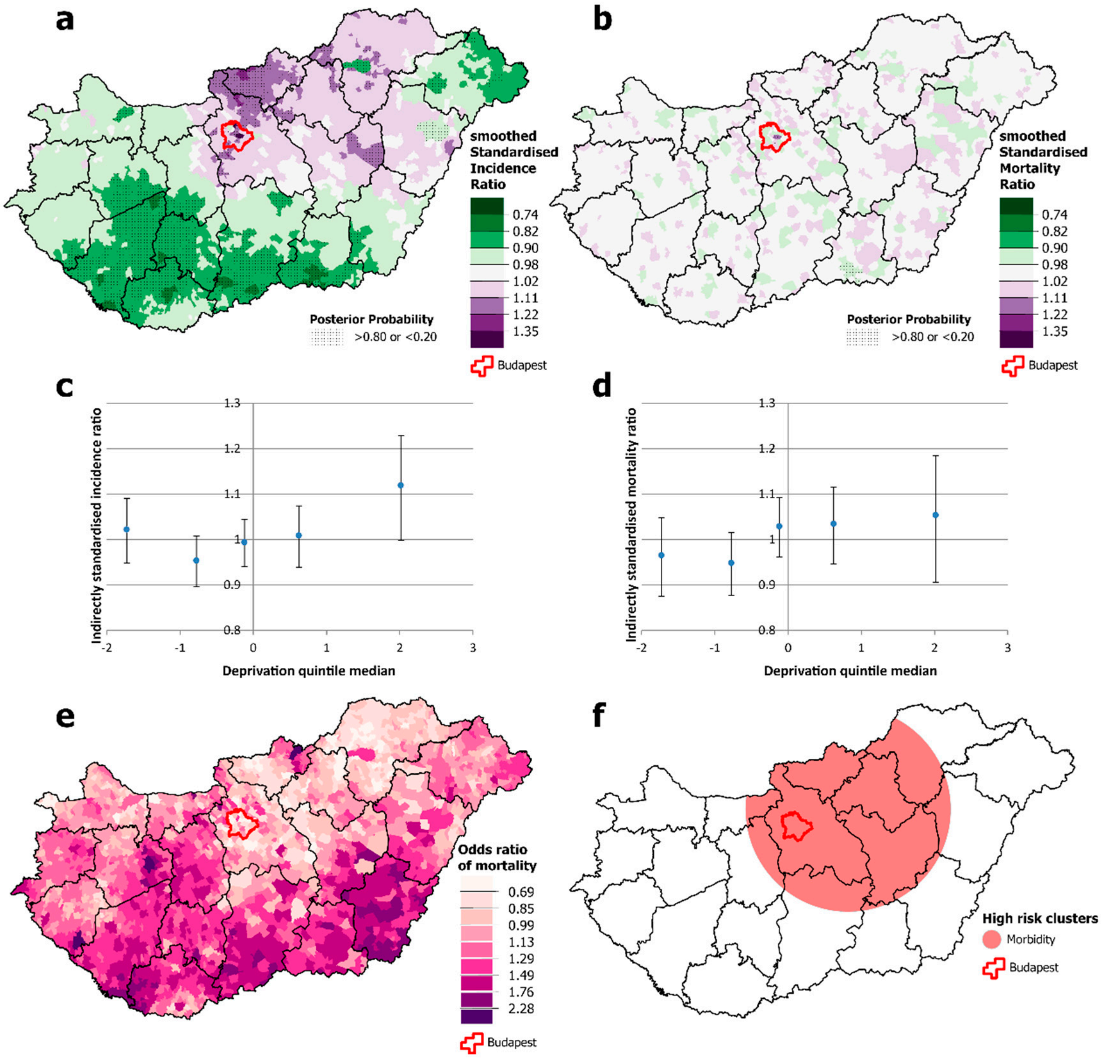


**Supplementary Figure S4**: Spatial distribution of incidence (a) and mortality (b); relationship between the deprivation and relative incidence (c) and mortality risk (d) by Deprivation Index quintile; odds ratio of mortality (e) and clusters of relative incidence, mortality (f) due to all malignant neoplasms of pancreas, aged 25-64 years, for females In Hungary, at municipality level, 2007-2018.
